# Spinal anesthesia with caudal catheter in pediatric urologic surgery: an alternative to general anesthesia

**DOI:** 10.3389/fped.2025.1614512

**Published:** 2025-11-05

**Authors:** Judith Stangl-Kremser, Kathleen Puttmann, Seth A. Alpert, Christina Ching, Daniel DaJusta, Molly Fuchs, Daryl McLeod, Venkata R. Jayanthi, Kristin Ebert

**Affiliations:** ^1^ Department of Urology, Ohio State University Wexner Medical Center, Columbus, OH, United States; ^2^ Department of Urology, Nationwide Children’s Hospital, Columbus, OH, United States

**Keywords:** pediatric urology, general anesthesia, spinal-caudal catheter anesthesia, complication, outcomes

## Abstract

**Introduction:**

To evaluate feasibility and outcomes of children undergoing complex urologic surgery who received spinal-caudal catheter (SCC) anesthesia compared to those who received general anesthesia (GA).

**Methods:**

A retrospective single-center analysis of children scheduled for urologic surgery under SCC anesthesia between 2016 and 2019 was performed. This group was compared with an age- and urologic procedure-paired GA cohort that included cases since 2010. Outcomes of interest included induction and operative times, intraoperative medication use, as well as anesthesia complications.

**Results:**

Each cohort was comprised of 52 patients. Induction times were longer with a mean difference of 4 min (*p* = 0.009) whilst operative times were shorter for the SCC group with a mean difference 34 min (*p* < 0.001). Mean intraoperative opioid dose was lower in the SCC group (0.014 vs. 0.19 MED/kg, *p* < 0.001). Fewer patients in the SCC group received corticosteroids (3.8% vs. 78.8%, *p* < 0.001). Complication rates did not differ significantly. There were two anesthesia related complications in the SCC group: transient myoclonic movement and retained catheter fragment; one GA case had intraoperative laryngospasm.

**Conclusion:**

SCC anesthesia is feasible in most patients undergoing complex urologic surgery as an alternative to GA. Although induction times are slightly longer, this may be worth the benefit, as regional anesthesia may reduce the need for using an airway device and intraoperative opioid use.

## Introduction

Almost 4 million surgeries are performed on children less than 18 years of age in the United States each year (1). A common parental concern is whether general anesthesia (GA) has potentially adverse effects on childrens' developing brains. This topic remains controversial. Neurotoxic effects of volatile general anesthetic agents have been demonstrated in animal studies (e.g., apoptotic neuronal cell death, decreased neuron density, and decreased neurogenesis); however, similar findings have been demonstrated for sedatives such as nitrous oxide and ketamine (2). In 2016, the FDA issued a safety announcement that prolonged exposure to GA agents may have negative effects on the developing brain (3). Of note, other intraoperative factors may influence neurocognitive growth, such as hypoxia and hypotension. Three large-scale studies (GAS, MASK, and PANDA) that were also included in a recent systematic review provide evidence that a single short GA exposure only has moderate risk of negative outcomes on cognition and is not associated with long-term neurodevelopmental abnormalities (4–7). Yet, use of multiple anesthetics and multiple exposures raises concern affecting neurocognitive function (6, 8, 9).

Anesthetic technique choice needs particular consideration especially in high-risk patients (10–12). GA with airway manipulation, as well as administration of opioids, can increase early postoperative apneic events. Regional anesthesia can decrease the need for opioid use, avoids airway management, and causes less perturbation of hemodynamics than GA (8). Our institution established a spinal anesthesia program for young children in 2016, which continues to operate successfully. Initial reports showed an 84% success rate of completion of surgery with this approach (13). However, it is generally limited to procedures that take less than 60–90 min, which is the limit of surgical anesthesia offered by spinal anesthesia. However, Jefferson et al. did demonstrate success in cases lasting a median of 95 min (14). Our institution designed a program where a spinal anesthetic is combined with placement of a caudal catheter, the spinal-caudal catheter (SCC), therefore allowing redosing of a neuraxial agent and aiming for use in longer and more complex procedures to be performed under regional anesthesia (15).

Although we have previously demonstrated feasibility of the SCC technique for urologic procedures in children (15), the benefits compared to GA remain uncertain. With this study, we aimed to compare outcomes (e.g., operative times, intraoperative medication use and anesthesia complication rates) of children undergoing SCC to those of GA for pediatric urologic procedures.

## Materials and methods

### Technique

SCC is typically discussed with families with children under the age of 4 years (1) who are interested in having their child undergo surgery under regional anesthesia and (2) who are having a surgery with incision at or caudal to the flank. The technique has been previously described by our group (15). In both groups (SCC and GA), at the discretion of the anesthesiologist, children received oral sedation with midazolam (0.5 mg/kg) preoperatively.

Our team's protocol for SCC is as follows: the lower back skin is numbed preoperatively with eutectic mixture of local anesthetics (EMLA) cream. Initial surgical anesthesia consists of a spinal block, placement of an intravenous line in the lower extremity, and then placement of a caudal epidural catheter. Patients are sedated intraoperatively with dexmedetomidine. One hour after the intrathecal injection with 0.5% bupivacaine (1 mg/kg), 3% chloroprocaine (1.5 ml/kg bolus followed by 1 ml/kg/hr infusion) is administered via the caudal epidural catheter to prolong the duration of surgical block (15, 16). 0.2% ropivacaine or 0.25% bupivacaine is administered in the post-anesthesia care unit via the caudal epidural catheter approximately 45 min after the chloroprocaine infusion is discontinued (15).

Our typical protocol for GA which was consistent in the GA group is that all patients undergo mask induction with inhaled general anesthetics, followed by placement of an intravenous line and an airway device (endotracheal tube or laryngeal mask airway). A caudal block is performed at the discretion of the anesthesiologist.

In both groups, intravenous corticosteroids (dexamethasone) were given at anesthesiologist discretion for patient comfort (e.g., anti-emetic prophylaxis) or if the patient was on chronic corticosteroids. Intravenous opioids were also given intraoperatively at anesthesiologist discretion for pain control.

### Data analysis

With approval by our Institutional Review Board (IRB #16-00249), a retrospective chart review of all children scheduled for surgery under SCC at our institution between 2016 and 2019 was performed. All children who successfully received SCC for surgery were included and compared to a control cohort who underwent GA. There were no strict inclusion/exclusion criteria for receiving SCC as a surgical anesthetic. Children unable to get a SCC were defined as failures and included local complications such as blood on initial placement of spinal needle, inability to pass cathether to adequate depth, and patient movement. These patients were excluded from the SCC group.

Baseline demographics were collected such as age, gender, weight and type of surgery. Surgical information included induction time, length of operative time (defined as time from incision to closure), out of the room time (defined as time from closure to leaving operating room), anesthesia complications, need for conversion to GA, and medication use (including sedatives, corticosteroids, and opoids). All subjects were paired with age- and procedure-paired controls who were retrospectively identified who received GA since 2010. For the procedure matching, some received an ancillary procedure along with the primary surgery, and an attempt was made to match those as well (i.e., if ureteral reimplantation was performed with diverticulum excision, we attempted to find a control who also had a diverticulum excision). For the age matching, all children undergoing SCC were within 6 months of their paired controls. Unless otherwise specified, data are presented as means (range or SD). Differences between groups were compared using a Chi square or Fisher's exact test for categorical variables and a Student's *t*-test for continuous variables. Data analysis was performed using SPSS Statistics software (IBM, version 24), and statistical significance was set at <0.05.

## Results

We identified 58 children who were scheduled for urologic surgery with SCC. Six of those (10.3%) were excluded due to inability to perform SCC and had to undergo GA. 5 excluded cases were identified prior to procedure start. They were ineligible due to failure of initial dural puncture for inthecal injection of spinal anesthesia (*n* = 4), and inability to pass caudal catheter to correct depth (*n* = 1). One patient moved during case (*n* = 1) and subsequently, the form of anesthesia had be converted to GA.

Eventually, the SCC group included 52 patients. Then, 52 age- and procedure-paired controls undergoing GA were identified resulting in a total cohort of 104 children. Baseline data of the overall cohort are outlined in Table 1. Surgical outcomes including induction and operative times are reported in Table 2. Interestingly, there was a small but statistically significant difference in mean induction time of 4 min (28.4 in SCC vs. 24.4 in GA, *p* = 0.009). The mean operative time differed by 34 min (112.8 vs. 146.8, *p* < 0.001). When comparing penoscrotal and abdominal/pelvic cases with SCC, there were no statistically significant differences in induction, operative, nor out of the room time after finishing with procedure. Preoperative sedation tended to be less frequent (*n* = 10 vs. *n* = 15, *p* = 0.050).

**Table 1:** Baseline data overall cohort (*n* = 104).

	Mean (range)
Demographics	
Age (months)	14.2 (2.4–46.7)
Weight (kg)	9.6 (3.5–17.9)
	*n* (%)
Male sex	
GA	33 (63.4)
SCC	36 (69.2)
Preoperative oral sedation	
GA	14 (26.9)
SCC	25 (48.1)
Anesthesia form	
GA	52 (50)
Endotracheal tube	42 (80.8)
Laryngeal mask	10 (19.2)
Caudal epidural block	44 (84.6)
SCC	52 (50)
Surgery performed with SCC	
Ureteral reimplant	14 (26.9)
1st stage hypospadias repair	12 (23.1)
2nd stage hypospadias repair	7 (13.5)
Pyeloplasty	5 (9.6)
Distal hypospadias repair	4 (7.6)
Single stage proximal hypospadias repair	4 (7.6)
Bilateral orchiopexy	2 (3.8)
Vaginoplasty	2 (3.8)
Ureteroureterostomy	1 (1.9)
Ureterostomy	1 (1.9)

GA, General Anesthesia; SCC, Spinal-Caudal Catheter.

**Table 2:** Surgical outcomes SCC vs. GA (*n* = 104) and PS vs. A/P (*n* = 52).

	SCC (*n* = 52)	GA (*n* = 52)	*p*
	Mean (SD)		
Induction time (minutes)	28.4 (8.6)	24.4 (6.4)	0.009
Operative time (minutes)	112.88 (26.35)	146.75 (54.27)	<0.001
Out-of-room time (minutes)	7.1 (4.1)	7.9 (4.4)	0.33
Intraoperative opioid use (MED/kg)	0.014 (0.05)	0.19 (0.17)	<0.001
	*n* (%)		
Preoperative sedation	25 (48.1)	15 (28.8)	0.044
Intraoperative corticosteroid	2 (3.8)	39 (78.8)	<0.001
Complication	2 (3.8)	1 (1.9)	1.0
	PS (*n* = 29)	A/P (*n* = 23)	
	Mean (SD)		*p*
Induction time (minutes)	28.58 (8.63)	28.13 (9.89)	0.8521
Operative time (minutes)	116.17 (27.73)	108.30 (24.74)	0.2920
Out-of-room time (minutes)	7.21 (3.46)	7 (4.90)	0.8595
Intraoperative opioid use (MED/kg)	0.017 (.056)	0.01 (0.03)	0.5170
	*n* (%)		*p*
Preoperative sedation	10 (34.48)	15 (65.22)	0.050
Intraoperative corticosteroid	0 (0)	2 (8.70)	0.191
Complication	2 (100)	0 (0)	0.497

A/P, Abdomen/Pelvis; GA, General Anesthesia; MED, Morphine Equivalent Dose; PS, Penoscrotal; SCC, Spinal-Caudal Catheter.

There were no significant differences in mean age (SCC 14.1 vs. GA 14.3 months, *p* = 0.90) and mean weight (SCC 9.7 vs. GA 9.6 kg, *p* = 0.88) between groups. Children in the SCC group were more likely to receive preoperative sedation with midazolam (SCC 48.1% vs. GA 28.8%, *p* = 0.044). The rate of intraoperative corticosteroid use was significantly higher in the GA group (SC 3.8% vs. GA 78.8%, *p* < 0.001). All children who received a corticosteroid in the SCC were on chronic corticosteroids for treatment of congenital adrenal hyperplasia. The mean opioid dose [in Morphine Equivalent Dose (MED)/kg] was significantly lower in the SCC group (SCC 0.014 vs. GA 0.19 MED/kg, *p* < 0.001) (Table 2).

Intraoperative complications were rare and not significantly different in both groups (*p* = 1.0) (Table 2). Two complications were seen in children undergoing SCC. One experienced myoclonic movements most likely due to local anesthetic toxicity. The chloroprocaine infusion was stopped, and the surgery was completed under the existing regional blockade. In another case, the tip of the caudal catheter sheared off on removal. One child in the GA group experienced an episode of laryngospasm during induction managed with positive pressure ventilation and intravenous propofol.

## Discussion

The use of combined regional techniques to avoid GA in children for longer urologic procedures is valuable and a useful goal. We were able to show that SCC is a feasible, lower-body anesthesia technique in young children undergoing complex urological procedures, with comparable outcomes to GA. The SCC group had longer induction (mean difference of 4 min), but shorter operative time (mean difference 34 min) but had lower requirements for intraoperative opioids. Anesthesia complication rate was not different between groups.

In our anecdotal experience, parents often have more concerns about anesthesia than the surgery itself, and may prefer to avoid GA. It is controversial if GA affects neurodevelopmental development (7), however it is generally considered to be safe (6, 7). However, duration and number of anesthesia as well as the dose of anesthetic agents should be limited. Patients with complex congenital conditions that affect the genitourinary tract may require many interventions under anesthesia and SCC can provide an opportunity to reduce repeated GA exposures in this population.

Regional anesthesia forms, as well as intraoperative sedation with dexmedetomidine, can also significantly decrease the requirements of opioids for surgical pain relief (17, 18). This has critical implications for patients in all age groups. infants are at particular risk for respiratory depression with opioids (19). Additionally, limiting opioids may allow for early ambulation. A recent analysis of 361 infants undergoing inguinal herniorrhaphy suggested a high safety level of regional anesthesia with benefits for early ambulation and pain control (8). The ERAS® pathway, which is aimed at maintaining physiologic homeostasis and minimizing surgical stress, includes opioid-sparing analgesia and early mobilization as fundamental pillars (20, 21). Following ERAS® principles has translated into reduced complications, length of stay, and costs while improving patient satisfaction (20). Use of the SCC technique aligns well with these principles.

SCC avoids the airway management of GA, which is particularly important in neonates who are at increased risk of serious airway complications (21). This decreased risk is particularly relevant for the performance of pediatric urologic procedures, which are primarily elective in nature. By keeping the infants awake, having them breathe spontaneously and protecting their own airway, common neonatal anesthesia concerns such as laryngospasm and increased airway resistance are removed (22). Our data demonstrate no airway complications in the SCC group and one airway complication in the GA group.

We did find that SCC anesthesia did come with a failure rate of 10.3%, comparable to a large multicenter study in the U.S (23). SCC anesthesia also introduced its own unique complications, including local anesthetic toxicity and complications with the catheter itself. These risks need to be discussed with families and balanced against the risks of GA.

Overall, current literature seems to suggest that regional anesthesia may have benefit for long procedures, and our SCC program was developed as a response to this data. This study is limited by its relatively small size and its single-institution and retrospective nature. We did not keep a separate database of general vs. general and caudal block. Moreover, there may have been patient selection, or internal time pressures in the surgeon, who may feel compelled to do the procedure faster because the patient is receiving regional anesthesia. In addition, we did not examine other important parameters relevant to regional anesthesia, such as effects on intraoperative hemodynamics or post-operative apnea rates. Hemodynamic issues are relevant to both regional and GA, with regional block causing less perturbation of hemodynamics than general anesthesia (8). Also, we did not specifically track post-operative apnea events, but think that this could be a meaningful outcome to evaluate in future work and could help clarify the true benefit of spinal anesthesia beyond traditional complication rates. Further, we do not have long term neuro-cognitive outcomes between these patient populations which would be interested as a follow-up study. Overall, we believe this cohort represents a valuable comparison between anesthesia types for urologic procedures, and that this technique merits further study.

## Conclusion

SCC with intravenous sedation is feasible in most pediatric patients as an alternative to GA with inhaled agents combined with caudal epidural anesthesia in complex pediatric urologic surgery. Potential advantages of SCC are no need for advanced airway management and decreased intra-operative opioid use with only a 4 min impact on induction times.

## Data Availability

The raw data supporting the conclusions of this article is available from the authors upon request.
